# Tuberculosis case fatality in India: a systematic review and meta-analysis

**DOI:** 10.1136/bmjgh-2019-002080

**Published:** 2020-01-20

**Authors:** Sophie Huddart, Anita Svadzian, Vaidehi Nafade, Srinath Satyanarayana, Madhukar Pai

**Affiliations:** 1 Epidemiology & Biostatistics, McGill University, Montreal, Quebec, Canada; 2 McGill International TB Centre, Montreal, Quebec, Canada; 3 International Union Against Tuberculosis and Lung Disease, Delhi, India

**Keywords:** tuberculosis, epidemiology, systematic review

## Abstract

**Introduction:**

The WHO End TB Strategy calls for a global reduction in the case fatality ratio (CFR) below 5%. India accounts for a third of global tuberculosis (TB) deaths. This systematic review estimated CFRs among Indian patients with TB both during and after treatment.

**Methods:**

We systematically searched Medline, Embase and Global Health for eligible studies published between 1 January 2006 and 8 January 2019, including both cohort studies and intervention study control arms that followed Indian patients with TB for fatality either during treatment or post-treatment. From relevant studies we extracted CFRs in addition to study demographics. Study quality was assessed using modified Scottish Intercollegiate Guidelines Network cohort criteria. Sufficiently homogenous studies were pooled using a random effect generalised linear mixed model. A meta-regression was performed to associate study characteristics with resulting CFRs.

**Results:**

218 relevant studies were identified, of which 211 provided treatment phase CFRs. Most patients (92.4%) were treated in the public sector. Quality concerns were identified in 74% of papers. We estimated a pooled treatment phase CFR of 5.16% (95% CI 4.20% to 6.34%) which fell to 3.78% (2.77% to 5.16%) when restricted to 52 high-quality studies. Treatment phase CFRs were higher for paediatric (n=27, 6.50% (2.65% to 10.36%)), drug-resistant (n=43, 14.06% (10.15% to 19.49%)) and HIV-infected (n=35, 10.91% (7.68% to 15.50%)) patients. Nineteen post-treatment CFR studies were too heterogeneous to pool except when restricting to three high-quality studies (2.69% (–0.79% to 6.18%)). Poor study quality (OR=2.27 (2.01 to 2.57)) and tertiary centres patients (OR=1.15 (1.03 to 1.28)) were significantly associated with increased treatment phase case fatality.

**Conclusions:**

Case fatality is a critical measure of the quality of TB care. While India’s treatment CFRs are in line with WHO targets, several key patient groups remain understudied and most studies suffer from methodological issues. Increased high-quality reporting on patient outcomes will help improve the evidence base on this topic.

**Key questions**

**What is already known?**

- India accounts for more than 25% of global tuberculosis (TB) incidence but there are concerns about the quality of care that patients receive.
- One of the WHO’s most important quality of care indicators is the case fatality ratio, with an ideal case fatality ratio below 5%.

**What are the new findings?**

- A systematic review of the literature yielded an overall case fatality ratio among Indian patients with TB during treatment of 5.16% (95% CI 4.20% to 6.34%).
- Case fatality was higher among key patient subgroups like those living with HIV or fighting drug-resistant TB.
- However, the quality of available studies was generally poor meaning that the literature estimates of case fatality may be biased.

**What do the new findings imply?**

- The TB field must better estimate case fatality with improved study design and statistical corrections for common biases.
- Special efforts must be made to monitor case fatality in the private sector and among patients who have completed treatment as current evidence for these groups is limited.

## Introduction

Tuberculosis (TB) affected 10.0 million new people in 2017 resulting in 1.6 million deaths globally.^1^ A key component to the WHO End TB Strategy^2^ is improving the quality of TB care.

The End TB Strategy calls for a 95% reduction in TB deaths by 2035 relative to 2015. One of the most important measures for quality of TB care is the case fatality ratio (CFR). At the country level, the CFR is estimated as the number of TB deaths divided by the number of incident cases in the same years, expressed as a percentage.^1^ In order to achieve the 2025 milestone of a 75% reduction in deaths, the End TB Strategy calls for the global CFR to fall from 15% to 6.5%.^2^ The WHO’s ideal global TB CFR is under 5%.^1^

India accounts for more than 25% of the global TB incidence.^1^ India has a complex healthcare system with a large private sector. Many patients with TB seek care from multiple providers before being diagnosed with TB and receiving appropriate treatment.^3^ Although India’s Revised National TB Control Programme (RNTCP) offers free TB therapy, over half of Indian patients with TB pay out-of-pocket to receive treatment in the unregulated private sector, where treatment quality often deviates from international standards.^4 5^ Publicly treated patients with TB are registered with the RNTCP and their treatment outcomes are recorded; however, no such routine treatment follow-up occurs in the private sector. In both the public and private sectors, no systematic post-treatment follow-up is conducted.

Globally, moderate quality data exist on patient fatality during TB treatment, mostly for publicly treated patients. A recent systematic review found a global CFR of 3.5% among patients who were HIV– and 18.8% among patients who were HIV+ of all ages.^6^ Of the Indian studies in this review, treatment phase case fatality ranged from 2.2% to 5.7%; these studies reflected only publicly treated patients.^6^ Globally, few studies estimate patient mortality after completing treatment. The available evidence suggests that patients with TB continue to experience significantly higher mortality after treatment when compared with the general population.^7^

In this systematic review, we summarise the available literature estimating treatment phase and post-treatment phase CFRs of Indian patients with TB and provide pooled CFRs among key subpopulations including HIV+, privately treated, drug-resistant and paediatric patients.

## Methods

This systematic review sought to estimate the treatment and post-treatment phase CFRs among Indian patients with TB after directly observed therapy (DOTS) scale-up in India (2006). A protocol with prespecified analyses was developed before conducting this review.

### Search strategy

Our search strategy focused on the intersection of concepts related to TB, death and India. The full search strategy can be found in online supplementary data S1.1.

10.1136/bmjgh-2019-002080.supp1 Supplementary data

On 8 January 2019, the Medline (1946–Present), Embase (1947–Present) and Global Health (1973–Present) databases were searched. We restricted to papers published in 2006 or afterwards to limit our data to the period where modern DOTS treatment was widely available across India.

Supplemental searches were conducted manually in the Indian Journal of Tuberculosis, Lung India and Indian Journal of Chest and Allied Diseases. A supplemental search was also conducted in the IndMed database. Additionally, we included programmatic data from RNTCP progress reports from 2007 to 2018.

### Outcome measure

A CFR is defined as the number of patients who die from any cause during the observed period divided by the number of patients forming the cohort at the beginning of the observed period. This differs slightly from the definition used at the country level and seen in the RNTCP reports as the number of incident cases does not need to be estimated; it is fixed by the design of the cohort.

Our primary outcomes were the CFR during the treatment phase and/or the post-treatment phase. The treatment phase was defined as the time period from treatment initiation to treatment completion or treatment cessation. The post-treatment phase was defined as the time period from treatment completion or cessation to the end of follow-up. If fatality data were not delineated between the treatment and post-treatment phase, an overall CFR was extracted.

### Eligibility criteria and study selection

We targeted prospective or retrospective cohort studies or control arms of intervention studies which described case fatality of any Indian patients with TB.

The specific inclusion criteria are as follows:

- Published on or after 1 January 2006.
- Covers, prospectively or retrospectively, Indian patients with TB after treatment initiation during either the treatment phase, post-treatment phase or both.
- Records case fatality during these phases.
- Cohort study or intervention study that allows a CFR to be estimated.

We excluded conference abstracts, study designs that did not allow for estimation of CFRs, study designs where patients were not randomly sampled (eg, case series), duplicate study populations and study populations where all patients with TB had the same comorbidity unless that comorbidity was HIV. We also excluded studies where the treatment phase follow-up did not begin at treatment initiation.

A title and abstract screen was performed independently by two reviewers (SH and VN). The full text screen was performed by SH and AS with disagreements resolved by consensus.

### Data extraction

Studies were extracted independently by SH and AS and then adjudicated. Extracted data included sample size, number of deaths and length of follow-up for the entire cohort and within any available patient strata in addition to cohort demographics and study quality data. The full list of extracted variables is available in online supplementary data S1.2 and the extracted data are available in online supplementary data S2.

10.1136/bmjgh-2019-002080.supp2 Supplementary data

### Quality assessment

Study quality was assessed using a modification of the Scottish Intercollegiate Guidelines Network cohort criteria.^8^ Because the included studies were descriptive cohorts and not intervention assessments, existing cohort evaluation tools were not completely suitable. Additional questions were adopted from ROBINS-I^9^ and Newcastle Ottawa Scale^10^ as appropriate. Major methodological concerns included cohort generalisability, selection bias due to loss to follow-up and appropriateness of length of follow-up. Studies were deemed to have poor generalisability if all patients were hospitalised or all patients had a rare form of TB (eg, TB of the ankle). Studies where more than 15% of patients were lost to follow-up were categorised as having a high risk of selection bias. Studies that followed patients for less than a month were categorised as having an inappropriately short follow-up. A study with one or more of the previous issues was classified as low quality. As a sensitivity analysis, low-quality studies were excluded from the meta-analyses.

### Meta-analysis methods

Case fatality estimates were pooled using a random-effects generalised linear mixed model (GLMM), which has been shown to outperform Der-Simonian and Laird models for meta-analysis of proportions because it exactly models the variance structure of binomial data.^11^ For each pooling, if the crude CFR (the sum of all deaths in all studies divided by the sum of the study sample sizes) was below 5% a Beta-Binomial GLMM was fit. If the crude CFR was above 5% a Normal-Binomial GLMM was fit. Beta-Binomial GLMMs have been shown via simulation to minimise error compared with Normal-Binomial GLMMs for rare events under 5%.^12^

While a forest plot was generated for each strata of interest, results were not pooled if there was substantial methodological or clinical heterogeneity in either the design or populations of the studies or if there is substantial statistical heterogeneity. As the more common I^2^ statistic is not available for GLMMs,^13^ statistical heterogeneity was assessed using τ^2^, a measure of interstudy heterogeneity.^14^ The decision to pool was made based on an assessment of clinic heterogeneity and a τ^2^<4. Values of τ^2^ are unique to each dataset and as such a universally applicable cut-off does not exist. For this work, a cut-off of four for τ^2^ was based on a holistic assessment of the range of τ^2^ across the strata and the precision of pooled CIs.

Treatment phase CFRs were pooled separately from post-treatment phase CFRs. In addition to the overall results, results within the following strata were examined: adult and paediatric patients, primary/secondary health centre and tertiary health centre patients, public and private sector patients, patients who were HIV+ and HIV– and drug-sensitive (DS) and drug-resistant (DR) patients. Studies with <2 patients in a given strata were excluded from the relevant strata pooling.

Routinely collected data on patient outcomes, including CFRs, are provided in annual RNTCP reports. The CFRs stratified by patient type are presented here from 2006 onwards; however, they are presented separately from the peer-reviewed literature and are not included in the pooled analyses. RNTCP reports were not included in pooling as they contain the data of many of the patients described in the included studies and thus would not be independent datapoints, a methodological requirement of meta-analysis. Additionally, they use the country level definition of CFR rather than the exact cohort definition used in the peer-reviewed studies.

### Meta-regression

A logistic meta-regression was fit for both the treatment phase and post-treatment phase studies with the relevant CFRs as the dependent variable. In order to not overfit the model, a limited number of study-level covariates were included in each model. Covariates were selected based on degree of missingness in order to maximise the number of studies which could be included in each meta-regression. For the treatment CFR meta-regression, those covariates were the proportion of patients with extrapulmonary TB (EPTB), treated in the private sector, living with HIV and with DR TB, as well as study quality (high or low) and study setting (primary/secondary centre or tertiary centre). The post-treatment CFR meta-regression included the proportion of patients with EPTB and study quality (High or Low). Model coefficients are presented as ORs. An example interpretation of an OR of 2 for study setting would be that the odds of case fatality are double for patient populations in tertiary centres compared with patient populations in primary and secondary centres, after adjustment for all other included variables.

Data analyses were performed in R (V.3.6.1) using the *metafor* (V.2.1) package and SAS (V.9.4M6).

### Patient and public involvement

This research was done without patient or public involvement.

## Results

**Figure 1:**
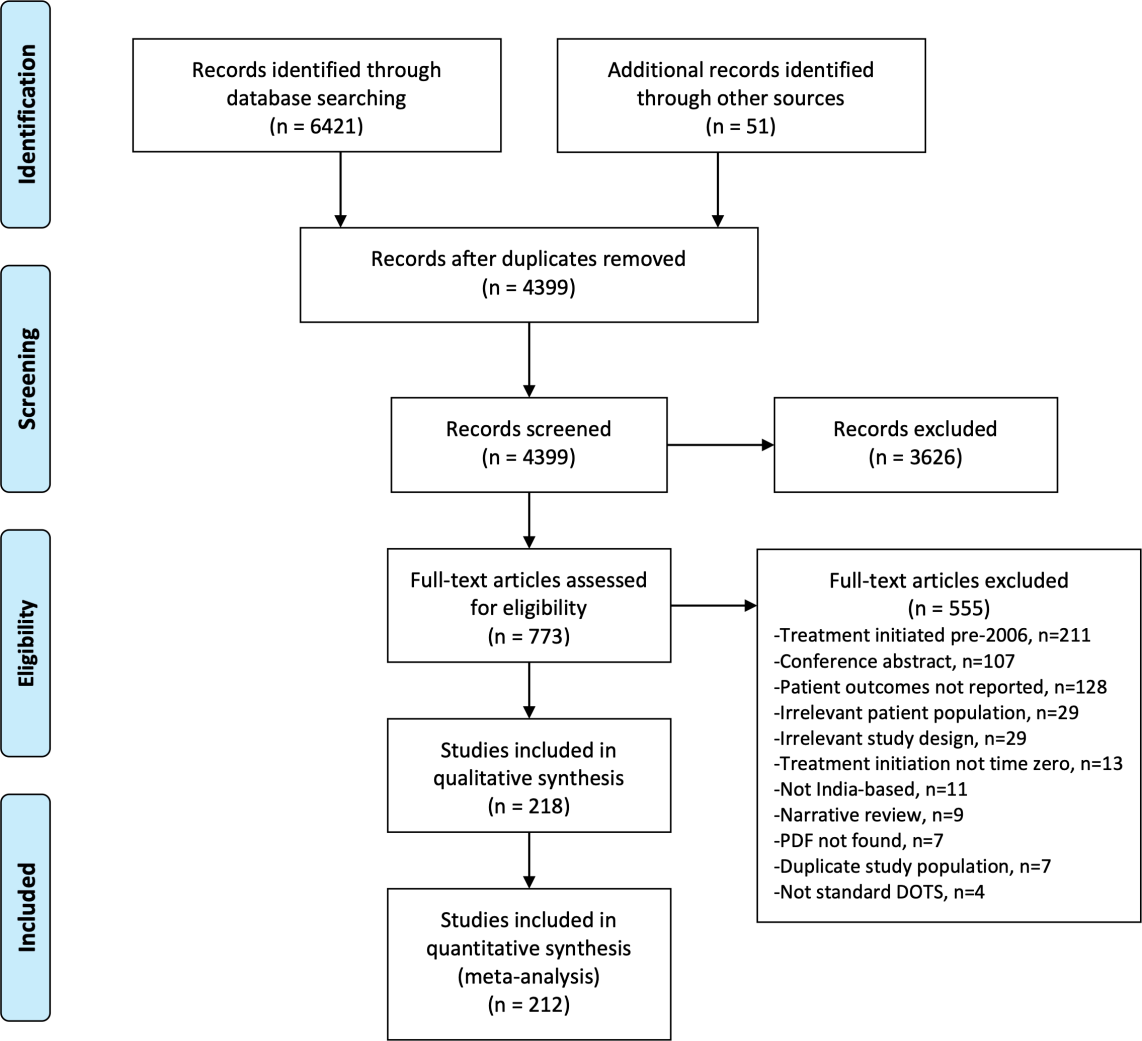
PRISMA flowchart of study selection.

Our search identified 4399 unique papers of which 733 full texts were screened. After screening, 218 relevant papers were identified (figure 1). Two hundred and eleven papers with treatment CFR information were included as well as 19 papers with post-treatment CFR information (one of which did not cover the treatment phase and thus is not included in the count of 211 treatment phase studies), for a total of 212 unique studies included in the quantitative analysis (full citations in online supplementary data S1.3). Six papers had the necessary information to calculate a CFR but did not delineate between treatment and post-treatment phases. These studies were not included in the quantitative analyses but can be viewed in online supplementary data S1.4.

**Figure 2:**
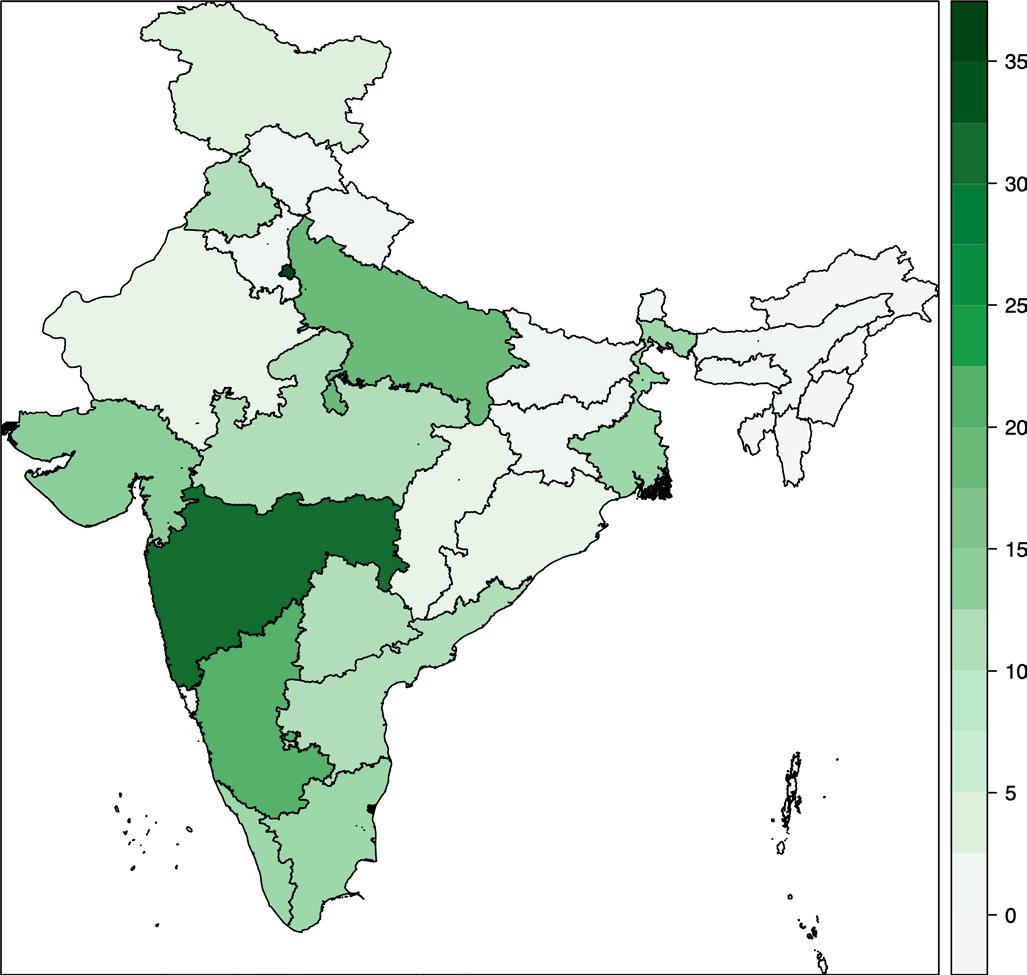
Heat map of included studies across Indian states. X-axis indicates the number of studies from each state.

The included studies provide good representation of the highly diverse^15^ Indian states (figure 2). About half the studies included patients from tertiary centres. With the exception of patient sex, level of the health centre and study location (which allowed for a public/private sector determination), critical patient demographics were often missing from studies (table 1). More than three quarters of studies failed to report the proportion of patients who received drug sensitivity testing and almost two thirds did not report the proportion of patients who were smear positive or the proportion of patients microbiologically versus clinically diagnosed.

**Table 1:** Summary of available study characteristics, n=218

	Average value	Per cent of studies not reporting demographic
Mean age (years) of patients included*	31.1	45.9
Per cent of studies in tertiary centres	55.5	0.0
Per cent <14 years old	18.5	42.7
Per cent female	52.9	8.3
Per cent people living with HIV	21.8	36.2
Per cent DR-TB	27.9	30.3
Per cent treated in the private sector	7.6	0.5
Per cent re-treated	33.7	48.6
Per cent EPTB	49.5	18.3
Per cent smear positive	54.7	60.1
Per cent microbiologically diagnosed	87.6	64.2
Per cent receiving DST	85.8	75.7

*Some studies reported median ages which are included in this average.

DR-TB, drug-resistant TB; DST, drug sensitivity testing; EPTB, extrapulmonary TB; TB, tuberculosis.

### Study quality

**Figure 3:**
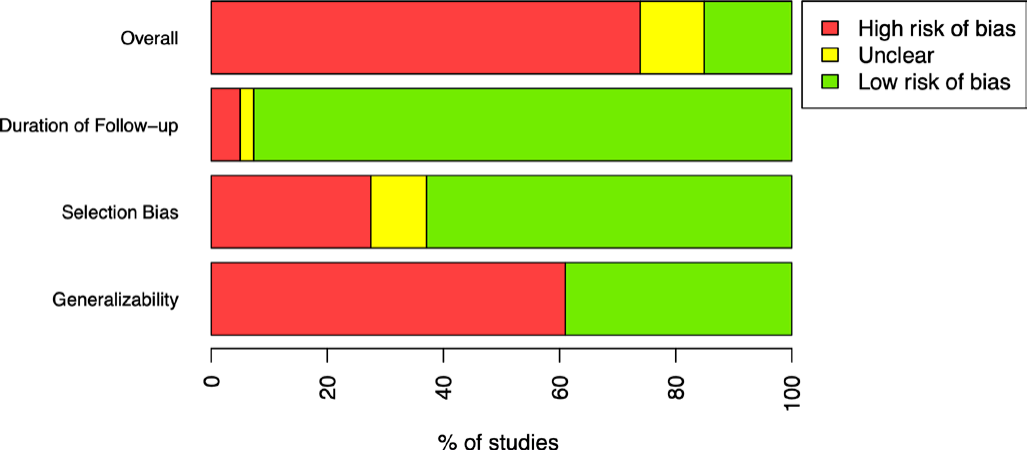
Summary of study quality assessment. If duration of follow-up was less than 1 month, studies were classified as having a high risk of bias. If more than 15% of patients were lost to follow-up, studies were classified as having a high risk of bias. If all patients were hospitalised or had a rare form of TB, studies were classified as having a high risk of bias. Unclear classifications indicate that insufficient information was available to assess these areas. If studies had a high risk of bias in any of the aforementioned areas, the study was classified as low quality. TB, tuberculosis.

A high risk of poor generalisability was found in 61.0% of papers and a high risk of selection bias was identified in 27.5% of papers. Finally, 5.0% of papers had follow-up periods too short to adequately capture fatality. Overall, 73.9% of papers were of poor quality for the reliable estimation of CFRs (figure 3).

### RNTCP reports

The RNTCP prepares annual reports of the previous year’s TB programme activity including treatment outcomes stratified by patient categories. The reports from 2007 (covering 2006 patient data) to 2018 (covering 2017 patient data) were included in this systematic review. Beginning in 2017, treatment outcome data was stratified by clinical and microbiological diagnosis status versus sputum smear status. Additionally, in 2011 the reports began to include multidrug-resistant (MDR) TB treatment outcomes and in 2013 HIV-TB specific treatment outcomes. The 2007–2016 report CFRs are available in table 2 and the 2017–2018 report CFRs are available in table 3. MDR TB and HIV-TB data from the 2011–2018 reports are available in table 4. The average CFRs for new smear positive (NSP), new smear negative (NSN) and new extrapulmonary TB cases were 4.1%, 3.7% and 2.6%, respectively (table 2). The average CFRs for new microbiologically diagnosed and new clinically diagnosed cases were 4.0% and 3.0%, respectively (table 3). Average CFRs for new HIV-TB, re-treatment HIV-TB and MDR TB cases were 13.0%, 14.4% and 21.0%, respectively (table 4).

**Table 2:** RNTCP report treatment CFRs during old classification system

	NSP	NSN	New EPTB	Smear positive re-treatment	New HIV-TB	HIV-TB re-treatment	MDR TB
2007	4.5	3.4	2.4	7.8			
2008	4.4	3.4	2.6	7.7			
2009	4.3	3.4	2.5	7.8			
2010	4.2	3.3	2.5	7.8			
2011	4	3	2	8			20
2012	4	4	2	8			18
2013	4	4	3	8	13	14	22
2014	4	4	3	8	13	14	22
2015	4	4	3	8	13	14	22
2016	4	4	3	8	13	14	22
Average	4.1	3.7	2.6	7.9	13.0	14.0	21.0

The significant digits appear here as they were reported by the RNTCP.

CFR, case fatality ratio; EPTB, extrapulmonary TB; MDR TB, multidrug-resistant TB; NSN, new smear negative; NSP, new smear positive; RNTCP, Revised National TB Control Programme; TB, tuberculosis.

**Table 3:** RNTCP report treatment CFRs with new classification system

	New—microbiological diagnosis	New—clinical diagnosed	Re-treatment—microbiological diagnosis	Re-treatment—clinical diagnosis	New HIV-TB	Re-treatment HIV-TB	MDR TB
2017	4	3	8	5			22
2018	4	3	8	4.8	13	16	20
Average	4.0	3.0	8.0	4.9	13.0	16.0	21.0

CFR, case fatality ratio; MDR TB, multidrug-resistant TB; RNTCP, Revised National TB Control Programme.

**Table 4:** RNTCP report treatment CFRs for HIV-TB and MDR TB across classification systems

	New HIV-TB	Re-treatment HIV-TB	MDR TB
2011			20
2012			18
2013	13	14	22
2014	13	14	22
2015	13	14	22
2016	13	14	22
2017			22
2018	13	16	20
Average	13.0	14.4	21.0

CFR, case fatality ratio; MDR TB, multidrug-resistant TB; RBTCP, Revised National TB Control Programme; TB, tuberculosis.

### Peer-reviewed literature

#### Treatment phase case fatality ratios

**Figure 4:**
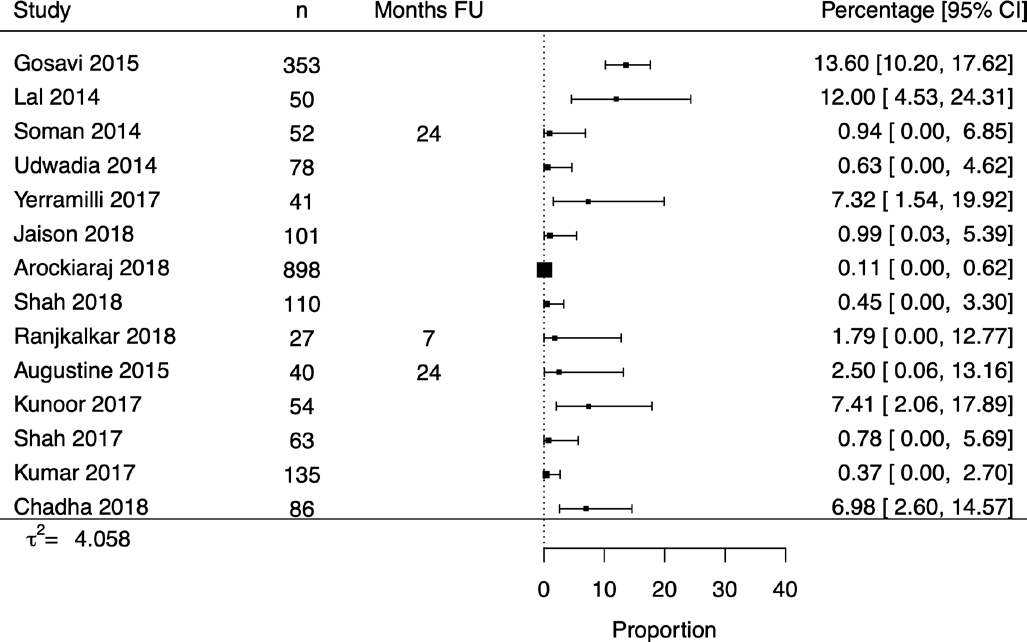
Forest plot of private sector treatment phase CFRs. CFR, case fatality ratio.

The 211 studies which described treatment phase CFRs had an overall pooled CFR of 5.16% (4.20% to 6.34%) (table 5). The paediatric pooled CFR (n=27) was 6.50% (2.65% to 10.36%) while higher CFRs were observed for patients with HIV infection (n=35, 10.91% (7.68% to 15.50%)) and DR-TB (n=43, 14.06% (10.15% to 19.49%)). The pooled treatment phase CFRs for primary/secondary centres (n=91, 5.18% (4.07% to 6.60%)) and tertiary centres (n=116, 4.87% (3.42% to 6.94%)) were similar. Fourteen papers with private sector CFRs were identified but their results were too heterogenous to reliably pool (figure 4).

**Table 5:** Treatment CFRs for all studies

	# studies	Pooled treatment CFR, % (95% CI)	τ^2^
Overall	211	5.16 (4.20 to 6.34)	1.961
Adult	73	4.62 (3.17 to 6.75)	2.155
Paediatric	27	6.50 (2.65 to 10.36)	3.463
HIV−	52	3.33 (2.00 to 5.52)	2.640
HIV+	35	10.91 (7.68 to 15.50)	0.900
Drug sensitive	104	3.58 (2.59 to 4.97)	2.389
Drug resistant	43	14.06 (10.15 to 19.49)	0.921
Public sector	193	5.40 (4.39 to 6.64)	1.811
Private sector	14	Not pooled	4.058
Primary/secondary centre	91	5.18 (4.07 to 6.60)	1.247
Tertiary centre	116	4.87 (3.42 to 6.94)	2.936

Private sector was not pooled due to high heterogeneity.

CFR, case fatality ratio.

#### Post-treatment phase case fatality ratios

The 19 studies which described post-treatment phase CFRs were more heterogenous than the treatment CFRs (table 6). Only the HIV-infected patient stratum was sufficiently homogenous giving a pooled post-treatment phase CFR of 4.15% (1.06% to 16.24%). There were substantially fewer studies that examined post-treatment fatality with only one study each providing post-treatment follow-up in the key populations of paediatric, DR and privately treated patients with TB.

**Table 6:** Post-treatment CFRs for all studies

	# studies	Pooled post-treatment CFR, % (95% CI)	τ^2^
Overall	19	Not pooled	5.214
Adult	11	Not pooled	4.041
Paediatric	1		
HIV−	4	Not pooled	0.000
HIV+	5	4.15 (1.06 to 16.24)	1.902
Drug sensitive	11	Not pooled	5.159
Drug resistant	1		
Public sector	19	Not pooled	6.042
Private sector	1		
Primary/secondary centre	9	Not pooled	4.265
Tertiary centre	9	Not pooled	4.868

Overall, adult, drug sensitive, public sector, primary/secondary centre and tertiary centre strata were not pooled due to high heterogeneity. The HIV− strata was not pooled as the model failed to converge.

CFR, case fatality ratio.

### Restricting to high-quality studies

#### Treatment case fatality ratios

Restricting to high-quality studies left 52 (52/211, 24.6%) studies concerning treatment phase CFRs (table 7). The overall pooled CFR reduced slightly to 3.78% (2.77% to 5.16%). The paediatric (n=11) CFR was substantially reduced to 1.08% (1.06% to 1.10%) while the HIV+ (n=7, 12.17% (5.68% to 26.11%)) and DR-TB (n=5, 11.78% (2.96% to 46.78%)) remained high. No high-quality private sector studies were identified. The high-quality study treatment phase CFRs were similar or slightly lower than the overall results with the exception of the HIV-infected strata, which increased slightly. All tertiary centre studies were excluded as low quality due to poor generalisability; thus, this stratum is not presented for the high-quality studies.

**Table 7:** Treatment CFRs for high-quality studies

	# studies	Pooled post-treatment CFR, % (95% CI)	τ^2^
Overall	52	3.78 (2.77 to 5.16)	1.132
Adult	14	4.34 (2.65 to 7.12)	0.782
Paediatric	11	1.08 (1.06 to 1.10)	3.463
HIV−	6	1.93 (0.86 to 4.38)	0.706
HIV+	7	12.17 (5.68 to 26.11)	0.782
Drug sensitive	34	3.94 (2.87 to 5.01)	0.739
Drug resistant	5	11.78 (2.96 to 46.78)	1.946
Public sector	52	3.78 (2.77 to 5.16)	1.132
Private sector	0		

#### Post-treatment case fatality ratios

Only three (3/19, 15.8%) high-quality post-treatment CFR studies remained after quality restriction though they were now sufficiently homogenous to pool for an overall post-treatment phase CFR of 2.69% (-0.79%, 6.18%). The three studies were all from the public sector. No high-quality post-treatment phase studies were available for paediatric patients, patients with DR-TB or patients with HIV-TB.

### Meta-regression

CFRs were regressed on study covariates for both treatment and post-treatment phase CFRs. There were 71 studies which had non-missing values for the required coefficients and a treatment phase CFR (table 8) and 19 studies which had non-missing values for the required coefficients and a post-treatment phase CFR (table 9).

**Table 8:** ORs from meta-regression of treatment CFRs, n=71

	Adjusted OR (95% CI)
EPTB (per 10% increase)	0.95 (0.94 to 0.97)
Private (per 10% increase)	0.86 (0.84 to 0.89)
HIV+ (per 10% increase)	1.15 (1.13 to 1.17)
DR TB (per 10% increase)	1.09 (1.08 to 1.10)
Tertiary setting (vs Primary/Secondary)	1.15 (1.03 to 1.28)
Poor quality (vs high quality)	2.27 (2.01 to 2.57)

CFR, case fatality ratio; DR TB, drug-resistant TB; EPTB, extrapulmonary TB; TB, tuberculosis.

**Table 9:** ORs from meta-regression for post-treatment CFRs, n=19

	Adjusted OR (95% CI)
EPTB (per 10% increase)	1.03 (0.98 to 1.07)
Poor quality (vs high quality)	0.75 (0.55 to 1.04)

CFR, case fatality ratio; EPTB, extrapulmonary TB; TB, tuberculosis.

For treatment phase CFRs, increasing proportions of EPTB (OR=0.95 (0.94 to 0.97)) and privately treated (OR=0.86 (0.84 to 0.89)) patients were significantly associated with lower odds of case fatality. Increasing proportions of patients with HIV infection (OR=1.15 (1.13 to 1.17)) and DR-TB (OR=1.09 (1.08 to 1.10)) were significantly associated with higher odds of case fatality. Studies set in tertiary settings were significantly associated with higher case fatality (OR=1.15 (1.03 to 1.28)) as was poor study quality (OR=2.27 (2.01 to 2.57)).

Post-treatment CFRs were not significantly associated with either proportion of patients with EPTB or study quality.

## Discussion

### Treatment phase

Our systematic review of the literature found an overall treatment phase CFR for Indian patients with TB of 5.16% (4.20% to 6.34%) among 211 papers. The pooled treatment phase CFR dropped slightly when restricted to high-quality studies to 3.78% (2.77% to 5.16%). Elevated treatment phase CFRs were identified for key patient subpopulations like those with HIV (10.91% (7.68% to 15.50%)) and those with DR-TB (14.06% (10.15% to 19.49%)). The paediatric TB CFR was found to be 6.50% (2.65% to 10.36%) in the full data but when restricting to high-quality studies it dropped to 1.08% (1.06% to 1.10%). In general, when restricting to high-quality studies, which were defined in part by having good generalisability to the entire TB population, pooled treatment phase CFRs were lower than the mixed quality pooled treatment phase CFRs. Generalisability concerns were the leading cause of declaring a study low quality suggesting that much of the available TB literature focuses on the sickest patients with TB like those who are treated in hospitals. This skewing towards sicker patients may be artificially elevating reporting of TB CFRs in the literature. Interestingly, the pooled treatment phase CFRs for primary/secondary health centres (5.18% (4.07% to 6.60%)) and tertiary health centres (4.87% (3.42% to 6.94%)) were similar but when adjusted for other study characteristics, tertiary centre studies were significantly associated (OR=1.15 (1.03 to 1.28)) with increased case fatality.

The overall pooled treatment phase CFR (5.16% (4.20% to 6.34%)) from the peer-reviewed studies since 2006 was higher than the average annual RNTCP reported CFR for NSP (4.1%), NSN (3.6%) and new EPTB (2.6%) cases over the same period. The pooled treatment CFRs for patients with HIV (10.91% (7.68% to 15.50%)) and DR-TB (14.06% (10.15% to 19.49%)) were lower than the RNTCP reported CFRs for these groups (new HIV-TB: 13.0%, re-treatment HIV-TB: 14.4%, MDR TB: 21.0%).

Our meta-regression associated pulmonary TB, public sector treatment, HIV positivity, drug resistance and tertiary health centre settings with increasing CFRs during treatment. Additionally, poor-quality studies were associated with finding higher CFRs.

### Post-treatment phase

The 19 papers that described post-treatment CFRs were highly heterogeneous and could only be reliably pooled when restricted to the three high-quality studies. The high-quality study post-treatment CFR was estimated to be 2.69% (–0.79% to 6.18%). Patient deaths after treatment may indicate either ineffective anti-TB treatment or a failure to address the socioeconomic determinants and physical environment that led to developing a disease like TB in the first place. TB treatment may also leave patients more susceptible to other diseases, both infectious and non-communicable. The goal of anti-TB treatment must extend beyond simply curing the current bout of TB to promoting long-lasting health for the patient.

### Extensive quality concerns

As discussed above, 61.0% of studies had poor generalisability due to hospitalised or other specialised patient populations. It is likely that these patients were sicker than the average Indian patients with TB and thus those studies had higher than representative CFRs. Many studies (27.5%) also had selection bias concerns. No study, including the RNTCP reports, corrected for patients lost to follow-up or those who transferred to other TB centres meaning that these patient outcomes are not reflected in the reported CFR. Patients lost to follow-up may have been lost because they had died which could bias reported CFRs downward. Overall, after adjusting for other study variables, poor study quality was associated with higher CFRs (OR=2.27 (2.01 to 2.57)) in our meta-regression.

### Strengths and limitations

The pooled overall treatment phase CFR estimated in this work is in line with the WHO End TB Strategy goal which is an important and positive step for India. However, key patient subpopulations are understudied or described in studies with potential biases. In more than a decade, only 14 studies have addressed case fatality during private TB treatment in India, a country where half of patients with TB are treated in the private sector.^4 5^ None of these 14 studies was of high quality. For all patient subgroups, post-treatment CFRs are understudied with only 19 studies identified. Support for patients with TB cannot stop when treatment is completed as patients are often in the same or worse social and environmental condition than when they first contracted TB.^16^ Study quality is also a major concern as almost three quarters of studies had potential biases. Our meta-regression suggested that studies with methodological issues were likely to find higher CFRs, potentially overestimating patient fatality.

Critically, this study focused on a WHO-identified key indicator of treatment quality: the CFR. This is a value monitored by TB programmes around the world and is relevant for programmatic planning. We were also able to include more than 200 studies thanks to flexible inclusion criteria that allowed for CFRs to be calculated from multiple study designs. Finally, our pooling methodology improved on the more common Der-Simonian and Laird models by exactly modelling the binomial variance of the CFRs and adapting as needed to rare events by using a Beta-Binomial GLMM.

However, it is important to keep in mind that the patients in the published literature are unlikely to perfectly represent the complete patient distribution of India. The literature likely over-represents rare forms of TB and hospitalised patients. We attempted to correct for this by restricting to high-quality studies that were evaluated for generalisability of the studied patient population. While we have reported on CFRs from the RNTCP government reports, we have excluded other grey literature that may exist. Additional data from the grey literature may have added power to this work but it is often difficult to assess the quality of non-peer-reviewed literature. Additionally, this work only reflects fatality during and after treatment. Studies that estimate case fatality before treatment initiation were not included; thus, we cannot speak to pretreatment CFRs. Similarly, the CFR is a measure of all-cause mortality and does not distinguish between TB or non-TB causes of death. In our meta-regression, which associated patient demographics and study characteristics with case fatality, we had to exclude many studies due to incomplete reporting of patient and study variables. Future studies on CFR and patient outcomes must fully report patient characteristics and patient selection. Moving forward, researchers and programmes must apply correction methods for patient loss to follow-up in order to minimise selection bias.^17^ Researchers should also recognise that hospitalised patients may systematically differ from most patients with TB and that only limited conclusions can be drawn from these populations.

Case fatality is a critical measure of the quality of TB care. While India’s overall treatment CFR is in line with WHO targets, several key patients groups remain understudied. Increased monitoring of patients treated in the private sector as well as follow-up of patients post-treatment will help ensure that all patients are able to achieve and maintain health after TB.
