# Supplementary material for: Tuberculosis case fatality in India: a systematic review and meta-analysis
Source: BMJ Glob Health. 2020 Jan 20;5(1):e002080. doi: 10.1136/bmjgh-2019-002080 (PMC7042607; doi:10.1136/bmjgh-2019-002080)
Supplement: Supplementary data [file bmjgh-2019-002080supp001.pdf]

S1. Supplemental Data

S1.1 Search Strategy

Medline search string follows. Search string was translated for each database.
(exp Tuberculosis/ OR tuberculosis.mp. OR TB.mp.) AND
(exp India/ OR india.mp. OR indian.mp.) AND
(mortality.mp. OR exp Mortality/ OR exp Death/ OR death*.mp. OR fatality.mp. OR CFR.mp. OR Follow-Up Studies/ OR follow*.mp. OR Prospective Studies/ OR Retrospective Studies/ OR exp Treatment Outcome/ OR treatment outcome*.mp. OR Survival Analysis/ OR Survival/ OR survival.mp.)

S1.2 Extracted Variables

First author last name
Study year
Source population
Setting of the study (in hospitals, tertiary care vs primary/secondary)
Study location (state)
Avg. patient age
Percentage <15 years old
Percentage female
Percentage HIV+
Percentage MDR or any drug-resistance
Percentage treated in public vs private sector
Percentage re-treatment
Percentage EPTB
Percentage Smear positive
Percentage Microbiologically Confirmed (from any source
Percentage urban
Patients received standard WHO DOTS? (Y/N)
Sample size (before loss to follow-up)
Average length of total follow-up
Maximum length of total follow-up
Person-years of follow-up
Number of patient deaths during entirety of follow-up
Overall case fatality proportion

{Average length of treatment follow-up
Maximum length of treatment follow-up
Person-years of treatment follow-up
Sample size of patient at beginning of treatment phase
Number of patients died during treatment phase
Number of patients with known outcome
Treatment case fatality rate
Average length of post-treatment follow-up

Maximum length of post-treatment follow-up
Person-years of post-treatment follow-up
Sample size of patient at beginning of post-treatment phase
Number of patients died during post-treatment phase
Number of patients with known outcome
Post-treatment case fatality rate}
{} Repeat again for HIV positive/negative, adult/child, public/private, drug sensitive/drug resistant as available

S1.4 Studies which did not delineate between treatment and post-treatment phase

Table S1.4.1: *Studies where CFR was not delineated between treatment and post-treatment*

	**N**	**Max follow-up (months)**	**CFR (95% CI)**
Sadana 2015	326	8	11.04 (7.95, 15.08)
Alvarez-Uria 2015	79	9	60.76 (49.1, 71.36)
Tutu 2017	115	12	0.87 (0.05, 5.45)
Sinha 2017	140	24	12.14 (7.44, 19)
Sinha 2017	31	12	6.45 (1.13, 22.84)
Gupta 2017	478	12	13.39 (10.53, 16.85)

S1.3 Table of included studies

Abbreviations: DMC – district microscopy center, RNTCP – Indian revised national TB program, MDR – multi-drug resistant TB, ATT – anti-tuberculosis treatment, MSF – Doctors without Borders, DR – drug resistant, EPTB-extrapulmonary TB, SS+ - sputum smear positive, TU – treatment unit, HAART – highly active antiretroviral therapy, NSP – new smear positive, TBM – TB meningitis, RCT – randomized control trial, DTC – district tuberculosis center, PHC – primary healthcare center, XDR – extensively drug resistant, NA – not applicable

	First Author's Last Name	Year	Title	State	Source Population	Total Patients Enrolled
1	Ahmad	2013	*Study Of Treatment Outcome Of New Sputum Smear Positive Tb Cases Under Dots – Strategy*	Maharashtra	Newly diagnosed smear positive TB patients in urban Mumbai slum treated at DMC	281
2	Akshata	2016	*Management of multidrug resistant tuberculosis (MDR-TB) – Monitoring is the key to successful outcome*	Karnataka	RNTCP-confirmed MDR TB patients treated at a Rajiv Gandhi Institute of Chest Diseases in Bangalore between Aug 2011 and June 2012	69
3	Alvarez-Uria	2012	*Natural History and Factors Associated with Early and Delayed Mortality in HIV-Infected Patients Treated of Tuberculosis under Directly Observed Treatment Short-Course Strategy: A Prospective Cohort Study in India*	Andhra Pradesh	all antituberculosis treatment (ATT) episodes of patients from the Vicente Ferrer HIV Cohort Study database from September 1st 2009, to September 1st 2011	1000
4	Ambadekar	2015	*Treatment outcome and its attributes in TB-HIV co-infected patients registered under Revised National TB Control Program: a retrospective cohort analysis*	Maharashtra	TB-HIV co-infected patients registered with RNTCP in Yavatmal district, from 2008 through 2011	886
5	Andries	2013	*High Rate of Hypothyroidism in Multidrug-Resistant Tuberculosis Patients Co-Infected with HIV in Mumbai, India*	Maharashtra	Patients initiated on a multidrug-resistant TB regimen between October 2006 and March 2013 at an MSF HIV project in Mumbai	116

6	Andries	2013	*Viral Load for HIV Treatment Failure Management: A Report of Eight Drug-Resistant Tuberculosis Cases Co-Infected with HIV Requiring Second-Line Antiretroviral Treatment in Mumbai, India*	Maharashtra	DR TB/HIV patients treated at MSF center in Mumbai between Oct 2006 and July 2013	8
7	Angadi	2016	*A Follow Up Study on Newly Detected Sputum Positive Pulmonary Tuberculosis Cases on Anti-Tubercular Treatment in Bijapur Taluk*	Karnataka	All newly detected pulmonary TB cases during November 1st 2011-October 31st 2012 registered from Bijapur Taluk at DTC, Bijapur	248
8	Anuradha	2010	*Predictors of stroke in patients of tuberculous meningitis and its effect on the outcome*	Uttar Pradesh	Adult patients with TB meningitis at outpatient and inpatient Medical University in Lucknow	100
9	Arathi	2013	*Osteoarticular Tuberculosis-A Three Years' Retrospective Study*	Uttar Pradesh	Osteoarticular TB patients diagnosed between Jan 2010 and Dec 1012 at a medical college in Uttar Pradesh	16
10	Armstrong	2014	*Treating drug-resistant tuberculosis in a low-intensity chronic conflict setting in India*	Andhra Pradesh-Chhattisgarh	DR TB patients treated as outpatients at MSF mobile clinic between Jan 2011 and Oct 2013	10
11	Ashwini	2009	*A Study on Treatment Outcome of Registered Tuberculosis Cases under RNTCP in Udupi Taluk, Karnataka*	Karnataka	TB patients registered under Udupi RNTCP between Jan and Dec 2007	563
12	Awasthi	2015	*Abdominal Tuberculosis: A Diagnostic Dilemm*a	NA	HIV- abdominal TB patients treated at a tertiary teaching hospital in N. India between Jan 2010 and Dec 2014	48
13	Babu	2012	*A Comparative Study Of Dot And Sat In Rural Tertiary Care Hospital: A Two Years Retrospective Study*	Karnataka	Pulmonary TB patients treated at a Karnataka hospital in 2010 and 2011	131

14	Bag	2015	*Resurgence Of Extra Pulmonary Tuberculosis*	Odisha	EPTB cases treated at a medical college between 2009-2013	2596
15	Banu Rekha	2012	*Efficacy of the 6-month thrice-weekly regimen in the treatment of new sputum smear-positive pulmonary tuberculosis under clinical trial conditions*	Tamil Nadu	Newly diagnosed SS+ HIV-pulmonary adult TB patients, above 30kg	249
16	Brahmapurkar	2016	*Death and defaulted trends among registered TB cases at Jagdalpur TU in Bastar district of Chattisgarh, India*	Chhattisgarh	TB patients registered at TU in Jagdalpur between 2010 and 2014	2533
17	Brahmapurkar	2016	*Treatment Outcome Of Registered Tuberculosis Cases For Year 2013 In Tuberculosis Unit In Tribal District Bastar Of Chhattisgarh, India*	Chhattisgarh	TB patients registered in 2013 at TU in Jagdalpur	468
18	Chandra	2017	*Factors Determining the Clinical Spectrum, Course and Response to Treatment, and Complications in Seronegative Patients with Central Nervous System Tuberculosis*	Karnataka	CNS TB patients seeking treatment at medical institute between Oct 2011 and Dec 2012	43
19	Chennaveerappa	2014	*TBDOTS Outcome in Relation to HIV Status: Experience in a Medical College*	Karnataka	Patients being treated at tertiary teaching hospital in South India	280
20	Daley	2015	*Adjunctive vitamin D for treatment of active tuberculosis in India: a randomised, double-blind, placebo-controlled trial*	Tamil Nadu	Participants (aged 18–75 years) who were HIV negative and had pulmonary tuberculosis, with at least one documented positive sputum smear, treated at RNTCP clinics, who had taken one dose of ATT or fewer	126
21	Dandekar	2014	*The Fate Of Tuberculosis Cases After Two Years Of DOTS Chemotherapy In Aurangabad City, Maharashtra*	Maharashtra	TB patients registered with RNTCP in Aurangabad in 2006	304

22	Das	2014	*HIV, multidrug-resistant TB and depressive symptoms: when three conditions collide*	Maharashtra	Adult MDR TB/HIV patients treated at MSF clinic in Mumbai between Aug 2012 and March 2014 with mental health assessment performed before treatment initiation	45
23	Dave	2015	*Direct Observation of Treatment Provided by a Family Member as Compared to Non- Family Member among Children with New Tuberculosis: A Pragmatic, Non-Inferiority, Cluster-Randomized Trial in Gujarat, India*	Gujarat	Children aged <15 years with newly diagnosed TB and registered for treatment under the RNTCP in Gujarat between June 2012 and Sept 2012	265
24	De	2011	*Incidence and risk factors of immune reconstitution inflammatory syndrome in HIV-TB coinfected patients*	West Bengal	Recently diagnosed HAART naive HIV-TB patients on ATT who started ART after 15 days of ATT, excluded non-adherent patients	96
25	Deepa	2013	*The Impact of Isoniazid Resistance on the Treatment Outcomes of Smear Positive Re-Treatment Tuberculosis Patients in the State of Andhra Pradesh, India*	Andhra Pradesh	Smear positive re-treatment TB patients without rifampicin resistance registered for treatment in three selected districts of Andhra Pradesh from April 2011 to March 2012 1077	
26	Baburao	2009	*Study of Tuberculosis cases under RNTCP attending Designated Microscopy Centre at Pravara Rural Hospital, Loni*	Uttar Pradesh	DMC patients registered from Jan 2006 to June 2008	611
27	Bhardwaj	2010	*Evaluation of adequacy of short-course chemotherapy for extraspinal osteoarticular tuberculosis using 99mTc ciprofloxacin scan*	Delhi	Spinal TB patients treated at tertiary hospital between Oct 2006 and Jan 2008	25

28	Chaudhri	2013	*Impact of psychiatric profile and personality trait on directly observed tuberculosis treatment outcome*	Uttar Pradesh	Adult new TB patients with psychiatric disorder diagnosed at DMC or initiated at DOTS center between Jan 2008 and Jan 2009, control arm of RCT on psychiatric intervention	88
29	Das	2016	*Initial Sputum Smear Grading Is A Predictor Of Smear Conversion And Treatment Outcome In Pulmonary Tuberculosis: A Retrospective Cohort Study*	West Bengal	SS+ patients registered at TU between Sep 2014 to Aug 2015	355
30	Dave	2013	*Assessment Of Long-Term Outcome Among New Smear Positive Pulmonary Tb Patients Treated With Intermittent Regimen Under Rntcp – A Retrospective Cohort Study*	Gujarat	Cured/Treatment completed NSP patients who finished between Jan and June 2009	706
31	Dave	2015	*Has introduction of rapid drug susceptibility testing at diagnosis impacted treatment outcomes among previously treated tuberculosis patients in Gujarat, India?*	Gujarat	All SS+ previously treated patients registered with RNTCP Jan-Jun 2013 in Gujarat	5829
32	Dewan	2014	*Early Outcome of Intermittent Directly Observed Treatment– Short Course, for Tuberculous Meningitis in Children: A Descriptive Analysis*	Delhi	Children with TBM admitted to medical college hospital between May 2008 and March 2009	30
33	Dhawan	2016	*Predictors of Neurological Outcome of Tuberculous Meningitis in Childhood: A Prospective Cohort Study From a Developing Country*	NA	Children with TBM at a research/teaching hospital in N. India	130
34	Dhingra	2009	*Tuberculosis Mortality Trends In Delhi After Implementation Of RNTCP*	Delhi	All Delhi patients registered with RNTCP in 2006	47608

35	Dholakia	2013	*Clinical profile and treatment outcomes of drug-resistant tuberculosis before directly observed treatment strategy plus: Lessons for the program*	Maharashtra	DR TB patients attending a TU in Mumbai between Aug 2006 and Nov 2010	29
36	Duraisamy	2014	*Does Alcohol Consumption during Multidrug-resistant Tuberculosis Treatment Affect Outcome?: A Population-based Study in Kerala, India*	Kerala	Culture-confirmed MDR TB patients treated under Kerala RNTCP between Jan 2009 and June 2010	179
37	Garg	2013	*Evaluation of prognostic factors in medically treated patients of spinal tuberculosis*	Uttar Pradesh	Spinal TB patients at Med. uni hospital July 2010 to Dec 2011	78
38	George	2011	*Predictors of mortality in patients with meningeal tuberculosis*	Kerala	TBM patients treated at government hospital between Jan 2006 and Dec 2008	98
39	Gohel	2016	*A study of 150 cases of intraperitoneal tuberculosis*	Gujarat	Intraperitoneal TB treated at hospital between 2006 and 2009	150
40	Gosavi	2015	*A Step Towards Control Of Multidrug Resistant Tuberculosis: Hospital Based Study From Nashik India*	Maharashtra	MDR TB cases treated at private hospital between Jan 2012 and March 2014	353
41	Goyal	2012	*Clinical and radiological prognostic indicators in childhood tuberculous meningitis*	Uttar Pradesh	Pediatric TBM patients treated at tertiary govt hospital between Sep 2010 and Aug 2011	53
42	Gupta	2015	*Spinal Cord and Spinal Nerve Root Involvement (Myeloradiculopathy) in Tuberculous Meningitis*	Uttar Pradesh	TBM patients diagnosed at government hospital between Nov 2011 and Nov 2013	71
43	Isaakidis	2012	*Adverse Events among HIV/MDR-TB Co-Infected Patients Receiving Antiretroviral and Second Line Anti-TB Treatment in Mumbai, India*	Maharashtra	MDR-TB patients who were initiated on ATT and ART between May 2007 and Sep 2011 at MSF clinic in Mumbai	67

44	Isaakidis	2011	*Ambulatory Multi-Drug Resistant Tuberculosis Treatment Outcomes in a Cohort of HIV-Infected Patients in a Slum Setting in Mumbai, India*	Maharashtra	HIV MDR TB patients treated at an outpatient MSF clinic in Mumbai between May 2007 and May 2011	58
45	Isaakidis	2015	*Outcomes in Adolescents Undergoing Treatment for Drug-Resistant Tuberculosis in Mumbai*	Maharashtra	Adolescent DR-TB patients treated at MSF clinic in Mumbai between July 2007 and April 2015	44
46	Isaakidis	2013	*Poor Outcomes in a Cohort of HIV-Infected Adolescents Undergoing Treatment for Multidrug-Resistant Tuberculosis in Mumbai, India*	Maharashtra	MSF treated teen MDR TB patients, Jul 2007 to Jan 2013	8
47	Iype	2011	*In-hospital mortality of intermittent vs daily antitubercular regimen in patients with meningeal tuberculosis - a retrospective study*	Kerala	TBM patients treated at medical college in Kerala between jan 2006 and Dec 2009	98
48	Iype	2014	*Major outcomes of patients with tuberculous meningitis on directly observed thrice a week regime*	Kerala	Adult TBM patients registered under RNTCP between Jan 2010 and Dec 2011 with Thwaites index 4 or below	47
49	Iype	2009	*Preliminary report of directly observed treatment, short course in tuberculous meningitis*	NA	TBM patients at medical college treated between Nov 2006 and Apr 2008	12
50	Jain	2010	*Outcomes of category III and I in immunocompetent patients of tuberculous lymphadenopathy treated in revised national tuberculosis control programme*	Madhya Pradesh	All Category I and III TB lymphadenopathy treated at hospital in Indore	295
51	Jain	2013	*Pediatric Tuberculosis in Young Children in India: A Prospective Study*	Maharashtra	Children under 5 with suspected TB treated at government hospital and associated clinic between Aug 2010 and Mar 2012	26

52	Jain	2011	*Profile of patients with gastrointestinal TB at a tertiary care centre in western India*	Maharashtra	Patients with abdominal TB treated at a Mumbai hospital between Jan and Dec 2009	32
53	Jain	2014	*Treatment outcome of standardized regimen in patients with multidrug resistant tuberculosis*	NA	All Category IV patients treated under RNTCP in an area in W. India between Jan 2009 and Dec 2009	130
54	Jha	2015	*Definite (microbiologically confirmed) tuberculous meningitis: predictors and prognostic impact*	Uttar Pradesh	Adult TBM patients at a tertiary hospital between Oct 2012 and Jan 2014	118
55	Joseph	2011	*Outcome of standardized treatment for patients with MDRTB from Tamil Nadu, India*	Tamil Nadu	DST confirmed adult MDR TB patients treated in Chennai RNTCP program between June 2006 and Sep 2007	38
56	Joseph	2011	*Treatment outcomes among new smear positive and retreatment cases of tuberculosis in Mangalore, South India – a descriptive study*	Karnataka	Category I (NSP) and II patients registered at Mangalore TU between June 2008 and May 2009	286
57	Kadarkar	2016	*Correlates of non-compliance to follow up sputum examination and treatment outcomes among tuberculosis patients under RNTCP in tribal area of Thane district*	Maharashtra	TB patients registered in Ganeshpuri DTC Jan to Dec 2013	126
58	Kalita	2014	*Predictors of paradoxical tuberculoma in tuberculous meningitis*	NA	Microbiologically confirmed TBM patients treated at a tertiary hospital between Aug 2009 and Feb 2012	34
59	Kalita	2014	*Safety and efficacy of levofloxacin versus rifampicin in tuberculous meningitis: an open-label randomized controlled trial*	NA	Adult new TBM patients treated at tertiary medical center between Jul 2009 and June 2012	60

60	Kapadia	2016	*Adverse Drug Reactions And Outcome Analysis Of MDR TB Patients On DOTS Plus Regimen*	Gujarat	MDR TB patients who completed treatment between Aug 2007 and June 2014 in Ahmedabad	102
61	Karmakar	2011	*Clinical Characteristics of Tuberculosis-Associated Immune Reconstitution Inflammatory Syndrome in North Indian Population of HIV/AIDS Patients Receiving HAART*	Delhi	HIV-TB ART-naive patients treated a Delhi hospital ART clinic	134
62	Kaur	2015	*Prospective Analysis of 55 Cases of Tuberculosis Meningitis (TBM) in North India*	Chandigarh	TBM patients treated in a tertiary referral centre between Aug 2010 and Feb 2011	55
63	Kumar	2016	*Prevalence and outcome of headache in tuberculous meningitis*	Uttar Pradesh	New HIV- TBM patients with headache attending tertiary hospital between Oct 2012 and March 2014	95
64	Lal	2014	*Study of gastro intestinal tuberculosis and role of surgery in its management in Navi Mumbai: analysis of 50 cases*	Maharashtra	Gastrointestinal TB patients treated at private hospital between Aug 2010 and Oct 2012	50
65	Lodha	2014	*Effect of micronutrient supplementation on treatment outcomes in children with intrathoracic tuberculosis: a randomized controlled trial*	Delhi	Pediatric intrathoracic TB patients in control arm of micronutrient RTC, Jan 2008 to Jun 2012	100
66	Lone	2014	*Impact of clinicoradiological parameters on the outcome of treatment in brain tuberculosis*	Jammu and Kashmir	Brain TB patients presenting at tertiary hospital between Nov 2009 and Nov 2011	61
67	Makharia	2015	*Intermittent Directly Observed Therapy for Abdominal Tuberculosis: A Multicenter Randomized Controlled Trial Comparing 6 Months Versus 9 Months of Therapy*	NA	Adult HIV- new abdominal TB patients at three tertiary centers	191

68	Malhotra	2013	*Corticosteroids (dexamethasone versus intravenous methylprednisolone) in patients with tuberculous meningitis*	Uttar Pradesh	Control arm of RCT in adult TBM patients treated at government hospital between Jan 2006 and Jul 2007	32
69	Mandal	2013	*Comparing the Daily Versus the Intermittent Regimens of the Anti-Tubercular Chemotherapy in the Initial Intensive Phase in Non-HIV, Sputum Positive, Pulmonary Tuberculosis Patients*	West Bengal	HIV- sputum positive pulmonary TB patients at tertiary care center between Jan 2010 and Dec 2011	83
70	Mishra	2007	*A study of effectiveness of DOTS on tuberculosis patients treated under RNTCP programme*	Madhya Pradesh	Patients registered at DOTS centres between Jul and Sep 2006 in Gwalior	312
71	Mukhopadhyay	2011	*Comparative Analysis of RNTCP Indicators in a Rural and an Urban Tuberculosis Unit of Burdwan District in West Bengal*	West Bengal	Patients at TU in 2007	898
72	Nagaraja	2011	*How Do Patients Who Fail First-Line TB Treatment but Who Are Not Placed on an MDR-TB Regimen Fare in South India?*	Andhra Pradesh	Re-treatment resistant RNTCP patients who were not given Category IV	202
73	Naik	2015	*Treatment Outcome of Patients with Extrapulmonary Tuberculosis on DOTS in a Teaching Hospital*	Karnataka	RNTCP treated EPTB patients diagnosed at private teaching hospital between 2010 and 2012	669
74	Nair	2016	*Impact of rapid molecular diagnostic tests on time to treatment initiation and outcomes in patients with multidrug-resistant TB, Tamil Nadu, India*	Tamil Nadu	MDR-TB patients in Tamil Nadu diagnosed with culture and sputum smear testing between Sep 2010 and Sep 2011	524
75	Nair	2017	*Predictors of unfavourable treatment outcome in patients with multidrug-resistant tuberculosis in India*	Kerala, Delhi and West Bengal	all patients enrolled for MDR-TB treatment in three states from Jan 2009 to Dec 2011	788

76	Nelliyanil	2012	*A Study Of The Socio-Demographic Profile And Treatment Outcome Of Paediatric Tuberculosis Patients In Bangalore Mahanagar Palike Area*	Karnataka	Pediatric patients registered in 2009	209
77	Padda	2015	*Treatment outcome of TB patients in a district of north India: a three year study*	Punjab	RNTCP patients registered between Jan 2011 and Dec 2013	2571
78	Panigatti	2014	*Profile and Outcome of Childhood Tuberculosis Treated with DOTS—An Observational Study*	Karnataka	Pediatric cases at government hospital receiving standard DOTS from Nov 2009 to Apr 2011	93
79	Parida	2014	*Comparison of Directly Observed Treatment Short Course (DOTS) with Self-Administered Therapy in Pulmonary Tuberculosis in Udupi District of Southern India*	Bangalore	Pulmonary TB patients diagnosed between Mar 2010 and Feb 2011, DOT arm of study	75
80	Patel	2016	*A Study Of The Timing Of Death In Patients With Tuberculosis Who Die During Anti-Tuberculosis Treatment*	Gujarat	All TB patients treated between Jan 2007 and Apr 2012 at charitable hospital	376
81	Patel	2016	*Treatment outcome among cases of multidrug-resistant tuberculosis (MDR TB) in Western India: A prospective study*	Gujarat	MDR patients registered at DOTS centre in govt hospital, Feb to Dec 2010	145
82	Pathan	2014	*HIV Serostatus And Treatment Outcome Among Patients Registered Under RNTCP*	Gujarat	TB patients registered with the RNTCP in 2009	395
83	Phageshwar	2013	*Incidence of Multidrug-Resistant (MDR) and Extensively drug Resistant (XDR) Tuberculosis among Different age Groups in Tertiary Care Hospitals of Chandigarh, India*	Punjab	Pulmonary TB patients registered to RNTCP at government hospital between Mar 2008 and Feb 2012	52

84	Piparva	2017	*Treatment Outcome Of Tuberculosis Patients On Dots Therapy For Category 1 And 2 At District Tuberculosis Centre*	Gujarat	Category I and II patients registered at DTC in 2013 and 2014	1340
85	Raizada	2009	*Linking HIV-Infected TB Patients to Cotrimoxazole Prophylaxis and Antiretroviral Treatment in India*	Andhra Pradesh	TB-HIV patients reported to RNTCP Mar to Aug 2007	734
86	Ramachandran	2017	*Factors Influencing Tuberculosis Treatment Outcome in Adult Patients Treated with Thrice-Weekly Regimens in India*	Tamil Nadu	Adult patients at RNTCP treatment centers from Oct 2013 to Sep 2015	1912
87	Ramachandran	2016	*Low Serum Concentrations of Rifampicin and Pyrazinamide Associated with Poor Treatment Outcomes in Children with Tuberculosis Related to HIV Status*	Tamil Nandu, Uttar Pradesh, Karnataka	Pediatric TB patients treated at hospital TB centres, 2010-2013	161
88	Ramzam	2013	*Childhood Tubercular Meningitis: An Institutional Experience and Analysis of Predictors of Outcome*	Kashmir	TBM ped patients treated at government hospital from June 2007 to May 2011	65
89	Rathee	2016	*Comparative study of clinico-bacterio-radiological pro le and treatment outcome of smokers and nonsmokers suffering from pulmonary tuberculosis*	NA	Adult pulmonary drug sensitive TB patients registered with RNTCP	101
90	Raut	2013	*Hydrocephalus in tuberculous meningitis: Incidence, its predictive factors and impact on the prognosis*	Uttar Pradesh	TBM patients at tertiary state university hospital treated from Oct 2010 to Aug 2012	80
91	Sadana	2015	*Socio-Demographic Factors Affecting The Treatment Outcome In Patients Of Tuberculosis*	Punjab	Category I, II and III patients registered at DTC between Apr 2010 and Mar 2011	326
92	Saha	2016	*Predictors of Treatment Outcome for Retreatment Pulmonary Tuberculosis Cases among Tribal People of an*	West Bengal	Retreatment tribal TB cases registered in TUs in 2013	177

			Eastern India District: A Prospective Cohort Study			
93	Samual	2016	*Relationship between Nutritional Support and Tuberculosis Treatment Outcomes in West Bengal, India*	West Bengal	SS+ PTB patients living below poverty line registered at TU between 2012 and 2013, some received nutritional support but not RCT	573
94	Sarpal	2014	*Treatment Outcome Among the Retreatment Tuberculosis (TB) Patients under RNTCP in Chandigarh, India*	Punjab	Retreatment cases registered under RNTCP between Jun 2010 and Dec 2011	545
95	Satyanarayana	2010	*Characteristics and Programme-Defined Treatment Outcomes among Childhood Tuberculosis (TB) Patients under the National TB Programme in Delhi*	Delhi	Pediatric TB patients registered with the RNTCP between Jan and June 2008	1074
96	Satyanarayana	2011	*Tuberculosis 'retreatment others': profile and treatment outcomes in the state of Andhra Pradesh, India*	Andhra Pradesh	Retreatment patients registered at TUs between Jul and Sep 2008	1009
97	Sharma	2015	*Clinicopathologic Spectrum of Cutaneous Tuberculosis: A Retrospective Analysis of 165 Indians*	Delhi	BCG vaccinated cerebral TB patients treated at tertiary public hospital	165
98	Sharma	2015	*Comparative Study of Laparoscopic Abdominopelvic and Fallopian Tube Findings Before and After Antitubercular Therapy in Female Genital Tuberculosis With Infertility*	Delhi	Female genital TB patients at tertiary public hospital	50
99	Sharma	2013	*Directly observed treatment, short course in tuberculous meningitis: Indian perspective*	Meghalaya	TBM patients at government tertiary hospital, diagnosed between Sep 2008 and Mar 2011	42
100	Sharma	2017	*Multi drug resistant female genital tuberculosis: A preliminary report*	Delhi	Female genital TB patients at tertiary hospital between Jan 2012 and June 2016	6

101	Sharma	2012	*Outcomes of Category III DOTS treatment in immunocompetent patients with tuberculosis pleural effusion*	Delhi, Andhra Pradesh, Gujarat, Rajasthan	Unilateral pleural effusion patients aged between 15 and 65 years treated as outpatients four tertiary hospitals across India between 2006 and 2011	351
102	Shastri	2013	*TB treatment outcomes among TB-HIV co-infections in Karnataka, India: how do these compare with non-HIV tuberculosis outcomes in the province*?	Karnataka	Programmatic data of TB and TB-HIV patients registered between Apr 2010 and Mar 2011	58446
103	Shibin	2014	*A Case Control Study on Clinical Characteristics And Treatment Outcomes In Tuberculosis Patients With Diabetes in Palakkad District*	Kerala	DTC adult TB patients enrolled Jan to Jul 2014	130
104	Shivam	2014	*Gender Differentials in Tuberculosis: An Experience from a Rural Tuberculosis Unit of Burdwan District, West Bengal, India*	West Bengal	Patients registered at TU between Nov 2010 and Dec 2011	758
105	Siddiqui	2016	*Effect of Diabetes Mellitus on Tuberculosis Treatment Outcome and Adverse Reactions in Patients Receiving Directly Observed Treatment Strategy in India: A Prospective Study*	Delhi	TB patients diagnosed at PHC Jan to Sep 2014	316
106	Singh	2015	*Clinicoradiological Profile of Lower Lung Field Tuberculosis Cases among Young Adult and Elderly People in a Teaching Hospital of Madhya Pradesh, India*	Madhya Pradesh	Adult (>12 yo) PTB patients treated at public tertiary hospital Jan 2012 to Jan 2015	215
107	Singh	2016	*Tuberculous lymphadenopathy: Experience from the referral center of Northern India*	Madhya Pradesh	TB lymphadenopathy patients (>12 yo) treated at a tertiary hospital between Jul 2013 and Jun 2014	204

108	Sinha	2012	*Early versus delayed initiation of antiretroviral therapy for Indian HIV-Infected individuals with tuberculosis on antituberculosis treatment*	Delhi	Adult newly diagnosed HIV-TB patients in RCT on the timing of ART initiation relative to ATT, took only delayed ART control arm, treated as outpatients at govt tertiary hospital	150
109	Sinha	2013	*Nevirapine versus efavirenz-based antiretroviral therapy regimens in antiretroviral-naive patients with HIV and tuberculosis infections in India: a pilot study*	Delhi	Newly diagnosed HIV-TB patients at tertiary hospital ART clinic between Sep 2007 and Dec 2012	135
110	Sinha	2011	*The antiretroviral efficacy of highly active antiretroviral therapy and plasma nevirapine concentrations in HIV-TB co-infected Indian patients receiving rifampicin based antituberculosis treatment*	Delhi	TB HIV patients at tertiary hospital diagnosed between Sep 2007 and Mar 2011	63
111	Soman	2014	*Successful Management of Highly Drug Resistant Tuberculosis with Individualised Drug Susceptibility Testing*	Maharashtra	MDR+ and XDR patients treated at a tertiary hospital	52
112	Swaminathan	2011	*Efficacy and Safety of Once-Daily Nevirapine- or Efavirenz-Based Antiretroviral Therapy in HIV-Associated Tuberculosis: A Randomized Clinical Trial*	Tamil Nadu	RCT of HIV drugs among adult newly diagnosed HIV/TB patients, took both arms	122
113	Thakor	2011	*Profile of paediatric TB cases in Ahmedabad Municipal Corporation area during year 2007 to 2009*	Gujarat	Pediatric patients treated at TU between 2007 and 2009	2253
114	Thomas	2011	*Extensively drug-resistant tuberculosis: experience at the Tuberculosis Research Centre, Chennai, India*	Tamil Nadu	MDR and XDR patients, 2006-2007 cohort	38

115	Tiwari	2012	*Relationship Between Sputum Smear Grading And Smear Conversion Rate And Treatment Outcome In The Patients Of Pulmonary Tuberculosis Undergoing Dots- A Prospective Cohort Study*	Delhi	Category I SS+ TB patients treated at DOTS centres between Nov 2006 and Oct 2007	338
116	Tripathi	2015	*Treatment Outcome Of Tuberculosis In Hiv Seropositive Patients: An Experience Of Southeast Region Of Ahmedabad*	Gujarat	HIV TB patients starting ATT at a medical college between Jan 2012 and Dec 2013	120
117	Tripathy	2013	*Effectiveness of a community-based observation of anti-tuberculosis treatment in Bangalore City, India, 2010–2011*	Karnataka	New SS+ RNTCP patients treated between Oct 2010 and Sep 2011	1864
118	Udwadia	2014	*Multidrug-resistant-tuberculosis treatment in the Indian private sector: Results from a tertiary referral private hospital in Mumbai*	Maharashtra	MDR TB patients treated at non-profit tertiary hospital between 2006 and 2010	78
119	Varshney	2011	*Incidence of various clinico-morphological variants of cutaneous tuberculosis and HIV concurrence: a study from the Indian subcontinent*	Uttar Pradesh	Cerebral TB patients treated at a private hospital between Oct 2007 and Nov 2009	131
120	Vashishtha	2013	*Efficacy and safety of thrice weekly DOTS in tuberculosis patients with and without HIV co-infection: an observational study*	Delhi	TB HIV patients initiating treatment at tertiary government hospital between Apr 2006 and Oct 2010	305
121	Vasudevan	2014	*Smear Conversion, Treatment Outcomes and the Time of Default in Registered Tuberculosis Patients on RNTCP DOTS in Puducherry, Southern India*	Puducherry	TB patients at TU between Jan and Jun 2011	660

122	Veeramani	2016	*Study on outcome of the treatment of tuberculosis patients registered under Revised National Tuberculosis Control Programme – DOTS strategy*	Tamil Nadu	TB patients registered at DOTS centers in 2014	282
123	Velayutham	2016	*Factors associated with sputum culture conversion in multidrug- resistant pulmonary tuberculosis*	West Bengal, Delhi and Kerala	DR TB patients treated by RNTCP between Jan 2009 and Dec 2011	787
124	Vijay	2011	*Treatment Outcome and Mortality at One and Half Year Follow-Up of HIV Infected TB Patients Under TB Control Programme in a District of South India*	Karnataka	TB-HIV patients registered with the RNTCP between Jul 2007 and Jun 2008	281
125	Viswanathan	2014	*Effect of diabetes on treatment outcome of smear-positive pulmonary tuberculosis— A report from South India*	Tamil Nadu	TB patients registered at TUs between Jan and Mar 2011	245
126	Shibin	2014	*A Case Control Study On Clinical Characteristics And Treatment Outcomes In Tuberculosis Patients With Diabetes In Palakkad District*	Kerala	DTC TB patients	130
127	Patel	2017	*A Randomized, Controlled, Phase III Clinical Trial to Evaluate the Efficacy and Tolerability of Risorine with Conventional Rifampicin in the Treatment of Newly Diagnosed Pulmonary Tuberculosis Patients*	Jammu, Bangalore, Ahmedabhad	Control arm of RCT	117
128	Saini	2016	*A Retrospective Cohort Study of Treatment Outcome among HIV positive and HIV negative TB patients in Chandigarh, India*	Punjab	RNTCP treated adult drug sensitive patients	3551
129	Misra	2016	*A study of hyponatremia in tuberculous meningitis*	Uttar Pradesh	TBM patients treated at government hospital	76

130	Marskole	2018	*A Study of Impact of CD4 and BMI on Effectiveness of DOTS in HIV-TB Cases Registered in Hamidia Hospital Bhopal*	Madhya Pradesh	TB and TB HIV patients in hospital DOTS and ART center	248
131	Das	2007	*A study of pulmonary tuberculosis in the elderly*	West Bengal	Pulmonary TB patients at hospital	70
132	Nagpal	2015	*A Study of Sputum Conversion Rates Affecting the Treatment Outcome in Newly Diagnosed Smear Positive Cases under DOTS in Amritsar City*	Punjab	NSP patients at TUs	250
133	Nelliyanil	2012	*A Study Of The Socio-Demographic Profile And Treatment Outcome Of Paediatric Tuberculosis Patients In Bangalore Mahanagar Palike Area*	Karnataka	TU pediatric TB cases	209
134	Gharat	2017	*A Study on Socio-demographic Profile and Outcome of Tuberculosis in HIV-TB Co-infected Cases in Surat City, Western India*	Gujarat	RNTCP HIV-TB patients	204
135	Yerramilli	2017	*A study on the clinical outcomes and management of meningitis at a tertiary care centre*	Telangana	Hospitalized TBM patients	41
136	Mukhopadhyay	2014	*Abdominal Tuberculosis with an Acute Abdomen: Our Clinical Experience*	West Bengal	Adult abdominal TB patients at government tertiary hospital between Jan 2009 and Dec 2011	70
137	Bunkar	2016	*Add-on prednisolone in the management of cervical lymph node tuberculosis*	Rajasthan	Cervical TB treated at hospital - DOTS only arm	60
138	Alvarez-Uria	2015	*Adding Streptomycin to an Intensified Regimen for Tuberculous Meningitis Improves Survival in HIV-Infected Patients*	Andhra Pradesh	All HIV-infected patients diagnosed with tuberculous meningitis from 1 January 2011 to 1 October 2014 from the Vicente Ferrer HIV Cohort Study database	79

139	Dela	2017	*Adverse drug reactions and treatment outcome analysis of DOTS plus therapy of MDRTB patients at district tuberculosis centre: A four year retrospective study*	Gujarat	MDR-TB patients treated by RNTCP, excluded natural causes of deaths	125
140	Kumar	2017	*An Overview Of Anti-Tuberculosis Treatment (ATT) In CAT I Newly Diagnosed Cases Of Tuberculosis In RNTCP*	Madhya Pradesh	Hospital outpatients, new drug sensitive TB	360
141	Puri	2017	*Antitubercular therapy induced liver function tests abnormalities in human immunodeficiency virus infected individuals*	Maharashtra	TB-HIV ATT-naive adult patients diagnosed with TB at ART centre	100
142	Kondapaka	2012	*Are Tuberculosis Patients in a Tertiary Care Hospital in Hyderabad, India Being Managed According to National Guidelines?*	Telangana	TB inpatients	1132
143	Dhoble	2017	*Assessment of the Socio-Demographic Profile and Treatment Outcome of Pediatric Tuberculosis Patients*	Maharashtra	Pediatric patients from TU	69
144	Jaison	2018	*Assessment Of Treatment Outcome In Tuberculosis Patients Approaching A Tertiary Care Teaching Hospital*	Kerala	TB patients treated at tertiary hospital	101
145	Datta	2015	Can Vitamin D Supplementation Hasten Recovery Of Tuberculosis	West Bengal	PTB hospital patients	271
146	Modi	2017	*Clinical and radiological predictors of outcome in tubercular meningitis: A prospective study of 209 patients*	Punjab	TBM patients at tertiary hospital	209
147	Synmon	2017	*Clinical and radiological spectrum of intracranial tuberculosis: A hospital based study in Northeast India*	Assam	Intracranial TB patients	93

148	Dole	2017	*Clinical Profile and Treatment Outcome of Drug Resistant Tuberculosis Patients of Western Maharashtra, India*	Maharashtra	DR TB patients treated at hospital	146
149	Mukherjee	2017	*Clinical Profile of Tubercular Empyema with Special Reference to Diagnostic Role of Cartridge Based Nucleic Acid Amplification test (CBNAAT)*	West Bengal	TB empyema patients at tertiary govt hospital	40
150	Mukherjee	2014	*Comparison between childhood and adult tuberculosis in a rural tuberculosis unit of West Bengal: A retrospective study*	West Bengal	Cases registered at TU between Jan 2008 and Dec 201, outcomes only for NSP	741
151	Parida	2014	*Comparison of Directly Observed Treatment Short Course (DOTS) with Self-Administered Therapy in Pulmonary Tuberculosis in Udupi District of Southern India*	Karnataka	Hospital DOTS records	75
152	Shukla	2016	*Comparison of Outcome of Dots and Self Administered Therapy in Patients of Tuberculosis in Tertiary Care Hospital, Nainital*	Uttarakhand	Hospital TB patients - DOTS arm	60
153	Gopalan	2018	*Daily vs Intermittent Antituberculosis Therapy for Pulmonary Tuberculosis in Patients With HIV A Randomized Clinical Trial*	Tamil Nadu	HIV-TB patients in RCT	98
154	Kant	2017	*Delay in initiation of treatment after diagnosis of pulmonary tuberculosis in primary health care setting: eight year cohort analysis from district Faridabad, Haryana, North India*	Haryana	Pulmoanry TB patients at PHCs	662
155	Koul	2016	*Demography and clinical outcome of pulmonary tuberculosis in Kashmir; 2 year prospective study*	Kashmir	Adult pulmonary TB patients at public hospital	72

156	Siddiqui	2017	*Diabetes prevalence and its impact on health-related quality of life in tuberculosis patients*	Delhi	DOTS centre TB patients	316
157	Das	2014	*Directly-Observed and Self-Administered Tuberculosis Treatment in a Chronic, Low-Intensity Conflict Setting in India*	Andhra Pradesh, Odisha	DOT arm of MSF treated patients, community healthcare centre, initiated in 2012	55
158	Arockiaraj	2018	*Drug resistant Skeletal Tuberculosis in a tertiary care centre in South India*	Tamil Nadu	DR skeletal TB at tertiary centre	898
159	Mittal	2011	*Effect of Disease Related Variables on Treatment Outcome Under DOTS*	Uttar Pradesh	DOTS patients	900
160	Kadam	2017	Effectiveness Of DOTS Therapy Under Rntcp-Dots Strategy In Paediatric TB-Experience From Western India	Maharashtra	Pediatric patients at tertiary hospital	104
161	Chopra	2017	*Efficacy of alternate day Directly Observed Treatment Short-course (DOTS) in skeletal tuberculosis – A retrospective study*	Delhi	skeletal TB at RNTCP clinic	218
162	Lanjewar	2014	*Evaluation of treatment outcome of tuberculosis patients in the urban field practice area of D. Y. Patil Medical College, Pimpri, Pune*	Maharashtra	Urban DOTS patients	429
163	Jain	2016	*Evaluation study of treatment outcome in Tuberculosis patients receiving DOTS under RNTCP*	Madhya Pradesh	RNTCP treated TB patients	241
164	Anandaraj	2017	*Factors influencing delay in sputum smear conversion among new smear-positive pulmonary tuberculosis patients of Davangere tuberculosis unit*	Karnataka	Adult pulmonary TB treated at DMC	233

165	Bhattacharya	2017	*Follow up of Drug-Resistant Pulmonary Tuberculosis Patients in a DRTB Center in First Fifteen Months of Treatment*	West Bengal	DR pulmonary TB patients at government hospital	142
166	Prasad	2016	*Frequency of adverse events observed with second-line drugs among patients treated for multidrug-resistant tuberculosis*	Uttar Pradesh	Pediatric TB cases	98
167	Taneja	2017	*Home Based Care as an Approach to Improve the Efficiency of treatment for MDR Tuberculosis: A Quasi-Experimental Pilot Study*	Delhi	Hospital chest clinic MDR patients	50
168	Mohammed	2018	*Hypothalamic and pituitary dysfunction is common in tubercular meningitis: A prospective study from a tertiary care center in Northern India*	Punjab	TBM patients at tertiary hospital	63
169	Mahmood	2018	*Impact Of Diabetes Mellitus On Treatment Outcome Of Multidrug Resistant Pulmonary Tuberculosis*	Uttar Pradesh	Hospital treated DOTS MDR patients	144
170	Bhatt	2018	*Impact of integrated psycho-socio-economic support on treatment outcome in drug resistant tuberculosis – A retrospective cohort study*	Delhi	Control arm in DOTS support trial at DTC	63
171	Iype	2011	*In-hospital mortality of intermittent vs daily antitubercular regimen in patients with meningeal tuberculosis–a retrospective study*	Kerala	TBM at tertiary hospital	55
172	Tutu	2017	Incidence And Pattern Of Adverse Drug Reactions (ADRs) In Patients Treated For Tuberculosis Under DOTS At A Tertiary Care Hospital Of Northern India	Uttar Pradesh	Hospital treated new TB DOTS patients	115

173	Shah	2018	*Increasing Prevalence of Pediatric Drug-resistant Tuberculosis in Mumbai, India, and Its Outcome*	Maharashtra	Pediatric DR-TB	110
174	Ranjkalkar	2018	*Isoniazid and rifampicin concentrations in children with tuberculosis with either a daily or intermittent regimen: implications for the revised RNTCP 2012 doses in India*	Tamil Nadu	Pediatric TB patients treated at tertiary hospital, intermittent DOTS arm	27
175	Mundra	2017	*Magnitude and determinants of adverse treatment outcomes among tuberculosis patients registered under Revised National Tuberculosis Control Program in a Tuberculosis Unit, Wardha, Central India: A record-based cohort study*	Maharashtra	Patients registered at TU in 2014	510
176	Jaryal	2011	*Manifestations of tuberculosis in HIV/AIDS patients and its relationship with CD4 count*	Himachal Pradesh	HIV-TB patients	87
177	Augustine	2015	*Multidrug-resistant tuberculosis: Need for better diagnostic modalities and clinical end points – Five-year tertiary care hospital experience*	Dehli	MDR culture confirmed patients at tertiary hospital	40
178	Sinha	2017	*Nevirapine- versus Efavirenz-based antiretroviral therapy regimens in antiretroviral-naive patients with HIV and Tuberculosis infections in India: a multi-centre study*	Delhi, Maharashtra	Efavirenz arm of HIV-TB RCT	140
179	Latief	2016	*Novel risk factors and early detection of anti tubercular treatment induced liver injury—Looking beyond American Thoracic Society Guidelines*	Jammu and Kashmir	TB patients treated at tertiary hospital	200

180	Sinha	2017	*Once Daily Dose of Nevirapine (400 mg) Versus Twice-Daily Dose (200 mg) of Nevirapine-Based Highly Active Antiretroviral Therapy Regimens in Antiretroviral Naïve Patients with HIV and Tuberculosis Infection in India*	Delhi	Control arm of HAART trial in TB-HIV patients	31
181	Kunoor	2017	*Outcomes of patients treated with individualised anti-tuberculosis regimens in a tertiary care centre in India*	Kerala	Patients treated at private tertiary hospital	54
182	Gupta	2017	*Predictors of adverse outcome in patients of tuberculous meningitis in a multi-centric study from India*	Delhi	TBM patients treated at hospital	478
183	Barman	2017	Profile And Treatment Outcome Among Pulmonary Tuberculosis Patients Under DOTS Attending A Tertiary Care Centre In Dibrugarh District, Assam	Assam	PTB patients diagnosed with PTB treated at tertiary hospital	661
184	More	2017	*Profile of drug-resistant tuberculosis in Western Maharashtra*	Maharashtra	Government treated DR TB patients	96
185	Shah	2017	*Profile of Tuberculous Cervical Lymphadenopathy in Children*	Maharashtra	Pediatric cervical lymph TB	63
186	Velayutham	2018	*Recurrence of tuberculosis among newly diagnosed sputum positive pulmonary tuberculosis patients treated under the Revised National Tuberculosis Control Programme, India: A multi-centric prospective study*	Tamil Nadu, Karnataka, Delhi, Maharashtra, Madhya Pradesh, Kerala	RNTCP new SS+ adult pulmonary TB patients	1565
187	Anandaraj	2017	Relationship Between Pre Treatment Bacillary Load And Smear Conversion And Treatment Outcome Of Pulmonary	Karnataka	Adult pulmonary TB patients treated at DMC	313

			Tuberculosis Patients In A TB Unit Of Karnataka			
188	Tiwari	2012	Relationship Between Sputum Smear Grading And Smear Conversion Rate And Treatment Outcome In The Patients Of Pulmonary Tuberculosis Undergoing DOTS- A Prospective Cohort Study	Delhi	Chest clinic DOTS patients	338
189	Kalita	2016	*Safety and efficacy of additional levofloxacin in tuberculous meningitis: A randomized controlled pilot study*	Uttar Pradesh	Control arm of TBM RCT	28
190	Sharma	2016	*Six months versus nine months anti-tuberculous therapy for female genital tuberculosis: a randomized controlled trial*	Delhi	Female genital TB with infertility	89
191	Rohit	2018	*Socio-demographic profile and outcome of TB patients registered at DTC Rewa of Central India*	Madhya Pradesh	DTC TB patients	133
192	Dhaked	2018	*Socio-demographic profile and treatment outcomes in pediatric TB patients attending DOTS centers in urban areas of Delhi*	Delhi	Pediatric TU patients in Delhi	140
193	Bagga	2017	Socio-Epidemiological Characteristics And Treatment Outcome Of Adult Tuberculosis Patients Under Directly Observed Treatment Short Course In Ludhiana City	Punjab	Adult TU patients	221
194	Lepcha	2017	*Sociodemographic profile and treatment outcome of tuberculosis patients registered under Directly Observed Treatment Short course in*	Sikkim	TU patients	2638

			East Sikkim with reference to defaulters			
195	Banga	2018	*Spinal Tuberculosis – Directly Observed Treatment and Short Course or Daily Anti Tubercular Therapy -Are We Over Treating?*	Punjab	Spinal TB treated at hospital - DOTS arm	30
196	Patel	2018	*Sputum Smear and Culture Conversion in Multidrug Resistance Tuberculosis Patients in Seven Districts of Central Gujarat, India: A Longitudinal Study*	Gujarat	DOTS Plus DR patients	142
197	Brahmapurkar	2017	*Sputum smear grading and treatment outcome among directly observed treatment short course patients of tuberculosis unit, Jagdalpur, Bastar*	Chhattisgarh	TU patients, 2014	496
198	Yadav	2016	*Study of factors influencing response and outcome of Cat-IV regimen in MDRTB patients*	Rajasthan	MDR patients, hospital DOTS+ centre	115
199	Patil	2017	Study Of Treatment Outcome Of Tuberculosis Patients Placed For Treatment Under Directly Observed Therapy Short Course (DOTS) At A Tuberculosis Unit, Karad, Western Maharashtra	Maharashtra	TU patients	319
200	Kumar	2017	Study Of Weight Variation During Anti-Tuberculosis Treatment In Tuberculosis Patients Put On DOTS In RNTCP In Central India	Madhya Pradesh	RNTCP treated patients at tertiary hospital	453
201	Singh	2018	*Surgery for Abdominal Tuberculosis in the Present Era: Experience from a Tertiary-Care Center*	Punjab	Abdominal TB at tertiary hospital	35

202	Kumar	2017	*Thoracoscopic Decortication of Stage III Tuberculous Empyema Is Effective and Safe in Selected Cases*	Delhi	TB empyema patients receiving surgery	135
203	Saharia	2015	*Thyroid profile status of patients treated for multidrug-resistant tuberculosis in state of Meghalaya, India*	Meghalaya	DTC MDR patients	114
204	Sarpal	2014	*Treatment Outcome Among the Retreatment Tuberculosis (TB) Patients under RNTCP in Chandigarh, India*	Punjab	RNTCP retreatment patients	545
205	Kumar	2018	Treatment Outcome And Efficacy Of Anti-Tuberculosis Treatment In Tuberculosis Patients Put On DOTS In RNTCP In Central India	Madhya Pradesh	Hospital outpatients starting DOTS	454
206	Cherian	2016	*Treatment outcome of extrapulmonary tuberculosis under Revised National Tuberculosis Control Programme*	NA	RNTCP EPTB patients in 3 districts	2046
207	Prajapati	2017	*Treatment Outcome of Patients Having Extensively Drug-resistant Tuberculosis in Gujarat, India*	Gujarat	XDR TB patients	112
208	Gupta	2018	*Treatment Outcomes Associated with Multidrugresistant Tuberculosis*	Delhi	MDR TB patients at tertiary DOTS center	819
209	Ramesh	2012	*Treatment outcomes of childhood tuberculosis with DOTS strategy in Kottayam, Kerala*	Kerala	pediatric TU patients	155
210	Prakash	2017	*Tuberculosis and human immunodeficiency virus co-infection: clinico-demographic determinants at an anti-retroviral therapy center in Northern India*	Bihar	HIV-TB patients at ART center	508

211	Chadha	2018	*Tuberculosis diagnostic and treatment practices in private sector: Implementation study in an Indian city*	Karnataka	Privately treated TB patients	101
212	Puri	2013	*Tuberculosis of the duodenum: clinical presentation, diagnosis and outcome*	Delhi	TB of the duodenum patients at tertiary hospital	10
213	Jackson	2017	*Tuberculosis treatment outcomes among disadvantaged patients in India*	Jarkkhand, Odisha, Chhattisgarh, Rajasthan, Maharashtra, Madhya Pradesh, Delhi	OpASHA treated patients	8415
214	Parmar	2018	*Unacceptable treatment outcomes and associated factors among India's initial cohorts of multidrug-resistant tuberculosis (MDR-TB) patients under the revised national TB control programme (2007–2011): Evidence leading to policy enhancement*	NA	all MDR patients registered with RNTCP 2007-2011	3712
215	Suryawanshi	2017	*Unfavourable outcomes among patients with MDR-TB on the standard 24-month regimen in Maharashtra, India*	Maharashtra	RNTCP MDR TB patients	4024
216	Dhuria	2016	*Universal access to DOTS in Delhi prisons: Where do we stand?*	Delhi	TB patients treated by RNCTP in Delhi prison	603
217	Dangeti	2018	*Vitamin D deficiency in patients with tuberculous meningitis and its relationship with treatment outcome*	Puducherry	TBM patients at tertiary hospital	39
218	Karthika	2018	*Why are people dying due to tuberculosis? A study from Alappuzha District, Kerala*	Kerala	RNTCP treated patients	1618
